# Integrated Analysis of Polyphenol Oxidase Gene Expression and Enzymatic Activity in Purple-Fleshed Potatoes

**DOI:** 10.3390/plants15071033

**Published:** 2026-03-27

**Authors:** Marilu Mestanza, Pablo Rituay, Angel David Hernández-Amasifuen, Dennis Eriksson, Alfonso H. del Rio, Jorge Alberto Condori-Apfata, Juan Carlos Guerrero-Abad

**Affiliations:** 1 Escuela de Posgrado, Programa Doctoral en Ciencias para el Desarrollo Sustentable, Facultad de Ingeniería Zootecnista, Biotecnología, Agronegocios y Ciencia de Datos, Universidad Nacional Toribio Rodríguez de Mendoza de Amazonas, Chachapoyas 01001, Peru; marilu.mestanza@untrm.edu.pe (M.M.); pablo.rituay@untrm.edu.pe (P.R.); 2 Instituto de Investigación, Innovación y Desarrollo para el Sector Agrario y Agroindustrial (IIDAA), Facultad de Ingeniería y Ciencias Agrarias, Universidad Nacional Toribio Rodríguez de Mendoza de Amazonas, Chachapoyas 01000, Peru; angel.hernandez@untrm.edu.pe (A.D.H.-A.); jorge.condori@untrm.edu.pe (J.A.C.-A.); 3 Department of Plant Breeding, Swedish University of Agricultural Sciences, SE-230 53 Alnarp, Sweden; dennis.eriksson@slu.se; 4 Department of Plant and Agroecosystem Sciences, University of Wisconsin—Madison, Madison, WI 53706, USA; adelrioc@wisc.edu

**Keywords:** enzymatic browning, gene expression, polyphenol oxidases (PPO), purple potato cultivars

## Abstract

Colored potato cultivars are rich in phenolic compounds that confer high antioxidant capacity; however, these beneficial metabolites could be susceptible to oxidation by polyphenol oxidases (PPOs), leading to enzymatic browning and the loss of antioxidant potential. Despite the agronomic relevance of this trade-off, the dynamics of the PPO gene family (*StPPOs*) gene expression in pigmented potatoes remains poorly characterized. Here, we present an integrated biochemical and molecular analysis of two purple-fleshed Peruvian landraces (Siriñacha and Angashungo), a partially pigmented landrace (Sapa), and non-pigmented cultivars, including the commercial cultivar Desirée. We quantified the total phenolic content, antioxidant capacity, and enzymatic browning index (EBI) using colorimetric and spectrophotometric methods. We also generated gene expression profiles of ten *StPPO* genes using semi-quantitative and digital PCR. Purple-fleshed cultivars exhibited significantly higher phenolic content and antioxidant capacity but also displayed accelerated browning kinetics compared to non- or partially pigmented genotypes. Expression analysis revealed cultivar-specific *StPPO* patterns, with *StPPO2* and *StPPO8* being markedly upregulated in pigmented materials, particularly *StPPO8*. These findings provide the first integrated biochemical and transcriptional evidence linking specific *StPPO* isoforms to enzymatic browning in colored potatoes, and highlight their potential for biotechnological applications.

## 1. Introduction

Potato (*Solanum tuberosum* L.) is one of most important staple crops [1] and is a significant part of Andean agrobiodiversity [2]. As the center of the domestication and diversification of the species, Peru harbors an exceptional reservoir of native cultivars that exhibit remarkable morphological and genetic diversity, as well as distinctive nutritional and functional properties [3].

Among native potato diversity, purple-fleshed cultivars are distinguished by their exceptional accumulation of phenolic compounds and anthocyanins [4], which have been associated with antioxidant and anti-inflammatory properties and favorable effects on cardiovascular and metabolic health [5]. These distinctive phytochemical effects confer value as functional foods and nutraceuticals, unlocking opportunities for accessing different national and international markets [6,7]. Their intense coloration and unique organoleptic properties make them potential components for value-added processed products [8]. However, these bioactive compounds with health-promoting properties may be affected by the action of polyphenol oxidases (PPOs) that results in enzymatic browning, an oxidative process that not only compromises the visual appearance of potato, but can also reduce its nutritional value by degrading bioactive compounds, such as anthocyanins, at the early stages of tuber processing or storage [9,10].

Enzymatic browning is one of the main reactions responsible for undesirable darkening in fresh plant products, particularly following exposure to oxygen, that takes place as after peeling or cutting [11,12]. This phenomenon is mediated by the action of polyphenol oxidases (PPOs), a group of oxidase enzymes that catalyze the oxidation of phenolic compounds to quinones, which subsequently polymerize to form dark-colored melanins [13].

At least ten genes encoding polyphenol oxidases (*StPPO1*–*StPPO10*) have been identified in the cultivated potato *Solanum tuberosum*, and their expression and activity vary according to tissue type as well as physiological stage and environmental conditions [14]. These copper-dependent PPOs are involved not only in enzymatic browning reactions, but also in defense mechanisms against pathogens [15]. The intensity of browning is determined by several factors, including the initial content of phenolic compounds and anthocyanins, oxygen availability, and the degree of tissue maturity [16,17].

Despite advances in the molecular characterization of polyphenol oxidases (PPOs) in commercial potato cultivars [14,18], the information available on pigmented cultivars, particularly those with purple flesh, remains limited. In these genotypes, it is still unclear which PPO isoforms and their gene expression in roots are associated with enzymatic browning in potato tubers. Hence, studies that integrate gene expression analysis with biochemical assessments of browning are needed to identify possible links between transcriptional regulation and PPO enzymatic activity in colored potatoes. The present study evaluated enzymatic browning in local purple-fleshed potato cultivars using a multifaceted approach combining biochemical and molecular analyses. Our results represent one of the first studies linking PPO gene expression with enzymatic browning in purple-fleshed cultivars and provide relevant information to understand the molecular mechanisms associated with this phenomenon. We expect this to be a starting point for additional research and for unlocking opportunities for biotechnological applications.

## 2. Results

### 2.1. Bioactive Compounds in Local Potato Cultivars

The Amazonas region in Peru consists of typical agroecosystems where small farmers maintain and crop important and diverse local potato cultivars. Many of these cultivars display a wide range of tuber flesh colors, including yellow, white, red, and purple, and are highly valued in local gastronomy. The local purple-fleshed cultivars Siricha and Angashungo were selected to evaluate their chemical composition in terms of phenolic content and antioxidant capacity.

Biochemical assays were performed on four local potatoes cultivars: Siricha and Angashungo (purple-fleshed), Sapa (partially pigmented) and Blanca (white fleshed), with the latter two included for comparison.

**Figure 1:**
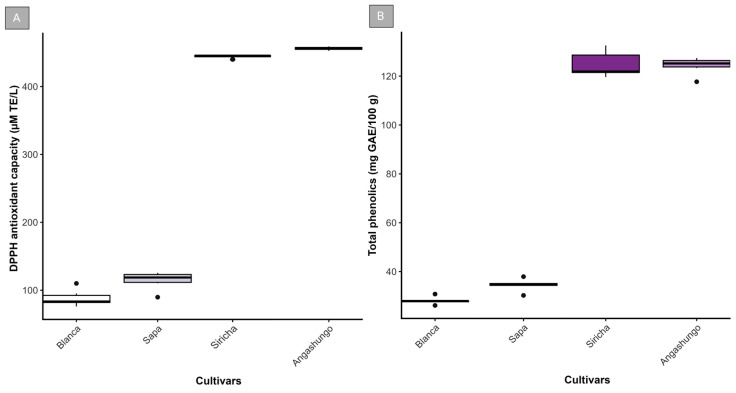
Antioxidant activity and total phenolic content in tubers of local potato cultivars. (**A**) Antioxidant activity determined by the DPPH assay. (**B**) Total phenolic content determined using the Folin–Ciocalteu method. Local cultivars included purple-fleshed (Siricha and Angashungo), partially pigmented (Sapa), and unpigmented (Blanca) potatoes. Values represent the mean ± SD of three biological replicates (*n* = 3), each analyzed with six technical replicates. Antioxidant activity is expressed as µM Trolox equivalents (TE) L^−1^ and total phenolic content as mg gallic acid equivalents (GAE) per 100 g fresh weight.

In the DPPH assay (Figure 1A), purple-fleshed cultivars exhibited the highest antioxidant capacity, with median values exceeding 450 µM TE/L and low variability among replicates. In contrast, Sapa and Blanca cultivars showed the lowest antioxidant capacity, with median values of approximately 120 and 90 µM TE/L, respectively. The total phenolic content (Figure 1B) was consistently higher in the purple-fleshed cultivars. Siricha and Angashungo presented concentrations close to 120–130 mg AGE/100 g, with limited dispersion; Sapa and Blanca exhibited lower values, with approximate medians of 35 and 28 mg AGE/100 g, respectively.

Both analyzed parameters showed a consistent pattern, with local purple-fleshed cultivars showing higher values than those observed in the partially pigmented and white cultivars.

### 2.2. Enzymatic Browning by Colorimetry

**Figure 2:**
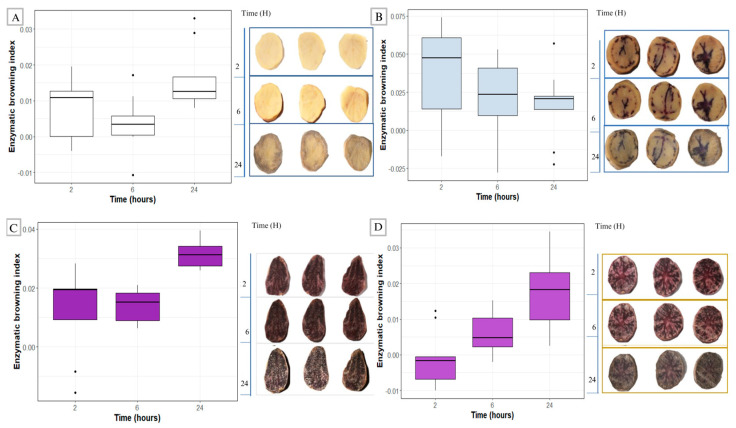
Enzymatic browning index determined by colorimetric assay in local potato cultivars at 2, 6 and 24 h after cutting. (**A**) Blanca, (**B**) Sapa, (**C**) Siricha and (**D**) Angashungo. The enzymatic browning index was calculated as the change (Δ) relative to the initial measurement (0 h). Values represent the mean ± SD of four biological replicates (*n* = 4), each analyzed in technical triplicate.

Colorimetry is a non-destructive method used to determine color changes on the surface of samples when subjected to specific treatment. We evaluated whether color changes occurred in purple-fleshed potato tubers after crosswise cuttings. Colorimetric measurements, after exposure, in potato slices at 2, 6, and 24 h (Figure 2) allowed calculations of the enzymatic browning index (EBI) over time. Purple-fleshed cultivars had relatively higher EBI values and a progressive trend in tissue darkening from the earliest evaluation times to the latest at 24 h (Figure 2C,D).

In contrast, the partially pigmented and white-fleshed cultivars exhibited lower values and moderate changes throughout the evaluation period. In these genotypes, darkening was less intense and progressed more slowly than in purple-fleshed cultivars (Figure 2A,B).

### 2.3. Chemical Evaluation of the Enzymatic Browning Index

**Figure 3:**
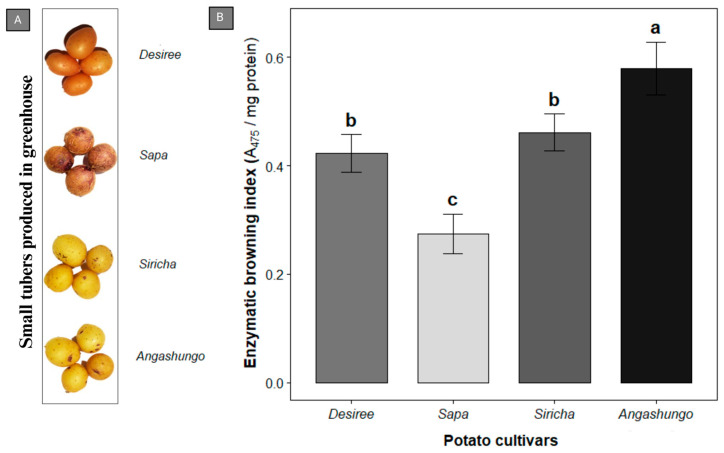
Mini-tuber morphology and enzymatic browning. (**A**) Morphological representation of mini-tubers obtained from in vitro-derived plants grown under greenhouse conditions. (**B**) Enzymatic browning index in local purple-fleshed potato cultivars. Values are expressed as the mean, ± standard deviation. The experiment was performed three times. Different letters above the bars indicate statistically significant differences among cultivars, as determined by one-way ANOVA followed by Tukey’s post hoc test (*p* < 0.05).

Chemical analysis allowed a direct method for quantifying enzymatic browning. As a complement to the colorimetric analysis, an independent chemical assay was conducted to validate the EBI values previously obtained. For this analysis, mini-tubers produced under greenhouse conditions from in vitro-derived plants were used to ensure a homogeneous physiological stage and development among the cultivars evaluated (Figure 3A).

A chemical analysis of the EBI revealed clear differences among the cultivars evaluated (Figure 3B). Angashungo exhibited the highest degree of browning, with values around 0.58 A_475_ mg^−1^ of protein, followed by Siricha, with values close to 0.46. In contrast, the partially pigmented cultivar Sapa displayed the lowest EBI value, at around 0.27, indicating reduced tissue darkening. Meanwhile, the white-fleshed cultivar Desirée showed intermediate browning intensity, with values around 0.42.

For this assay, the cultivar Desirée was included as a reference cultivar, given its extensive use as an experimental standard in studies of enzymatic browning and gene expression. This variety replaced the local, white-fleshed cultivar previously evaluated in the colorimetric assay.

All of these results revealed an enzymatic browning gradient, with higher values observed in purple-fleshed cultivars, a pattern consistent with those previously detected using colorimetric analysis.

### 2.4. Differential Expression of StPPO Genes in Roots of Purple-Fleshed Potato Cultivars

Given that both colorimetric and chemical analyses revealed greater enzymatic browning in local, purple-fleshed cultivars, we then evaluated the expression of polyphenol oxidase genes in potato roots under in vitro conditions. Polyphenol oxidases are encoded by a multigene family previously described in the *Solanum tuberosum* genome. Chi et al. [14] reported the presence of ten *StPPO* genes potentially involved in this process. In this context, the expression profiles of these genes were analyzed in the roots of local cultivars and the reference cultivar Desireé to identify transcriptional differences. For this purpose, we use semi-quantitative PCR and digital PCR (dPCR), with the latter used to quantify copies of cDNA [19,20].

**Figure 4:**
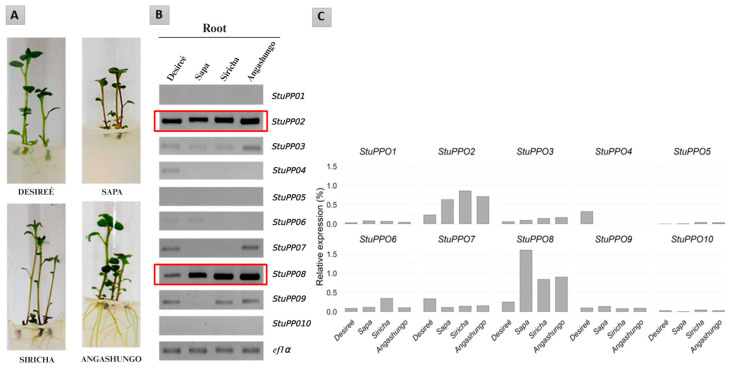
Gene expression analysis of *StPPO* genes in roots of three local potato cultivars and Desirée. (**A**) *In vitro*-grown potato plants at 25 days of growth were used for the gene expression analyses. (**B**) Semi-quantitative PCR analysis of *StPPO1*–*StPPO10* expression in roots, performed for 28 cycles. Red boxes show genes more differentiated expressed in roots. (**C**) Quantitative analysis by digital (dPCR), performed for 40 cycles. Gene expression experiments were performed in duplicate and *ef1α* was used as the reference gene. Panels (**B**,**C**) represent independent methodological approaches and are not intended as directly quantitative equivalents.

Gene expression analysis revealed differential transcription patterns among the cultivars. Semi-quantitative PCR found that *StPPO2* and *StPPO8* exhibited the highest expression levels in root tissue with consistent signals across all materials analyzed, including Desirée (Figure 4B). In contrast, the remaining members of the *StPPO* gene family showed weak or undetectable expression.

Quantification by digital PCR (dPCR) confirmed these findings (Figure 4C). *StPPO2* and *StPPO8* showed the highest levels of transcription, whereas the remaining genes exhibited low expression levels or values close to the detection limit. Notably, *StPPO8* displayed higher expression in purple-fleshed cultivars than in Desirée, indicating a preferential expression associated with these genotypes.

## 3. Discussion

The local purple-fleshed potato cultivars analyzed in this study showed a higher content of phenolic compounds and greater antioxidant activity compared to the white-fleshed cultivar used as a reference. This pattern is consistent with previous studies reporting a greater accumulation of phenolic compounds and anthocyanins in pigmented potatoes compared to non-pigmented cultivars [21,22,23]. For example, Silveira et al. [24] observed increases ranging from 1.5 to 3 times more total phenolic content in purple-fleshed cultivars compared to white-fleshed cultivars. The differences observed in our study were comparable, suggesting that the local cultivars evaluated maintained the characteristic of metabolic profile described for pigmented potatoes. These compounds have been extensively associated with functional activity and health benefits attributed to purple-fleshed potatoes [4,5,25].

From a biochemical perspective, this finding is relevant because phenolic compounds are direct substrates of PPOs, key enzymes involved in enzymatic browning [13,26]. Consequently, the greater availability of phenolic substrates may promote more intense oxidative reactions following tuber cutting.

Colorimetric browning assays revealed higher levels of oxidation in purple-fleshed cultivars than in partially pigmented and white cultivars, as reported by Silveira et al. [24], where greater susceptibility was observed in a purple cultivar named “Bruja” when compared with less pigmented and white cultivars. It is important to note the limitations of the colorimetric approach, because the colorimetric data values can be affected by starch release and the effect of this on surface gelatinization [27,28]. Consequently, white-fleshed cultivars may show lower oxidation because of their higher starch content as compared to pigmented cultivars [29]. Because of these limitations, a chemical analysis of the enzymatic browning index (EBI) was also used in our experiments since this approach is able to measure the specific enzymatic activity of browning [18].

We performed the analysis of the EBI using mini-tubers at early developmental stages, characterized by incomplete pigmentation [30], and potential phenotypic variations associated with acclimatization [31,32]. These conditions did not alter the overall trend because the responses of the genotypes were assessed under the same physiological conditions, and the response for the EBI in local cultivars maintained higher browning values than the reference cultivar Desirée, which has been widely used in experimental studies [14,18,33].

Given this increased susceptibility to browning, the underlying molecular factors were further investigated. In *Solanum tuberosum*, a multigene family composed of ten *StPPO* genes with differential expression patterns has been described [14]. Among these, *StPPO1*–*StPPO4* correspond to the predominant isoforms in tubers and roots, with *StPPO2* identified as the main contributor to enzymatic activity in the reference cultivar [33,34,35]. Our gene expression analysis using semi-quantitative PCR and digital PCR resulted in identifying the latter as the one providing high accuracy and reproducibility in detecting quantitative gene expression [36,37,38] and revealed high transcription levels of *StPPO2* in root tissue for all genotypes. In fact, the editing of the *StPPO2* gene significantly reduced tuber darkening [18], supporting its central role in this process. The recurrent high expression levels observed in our local cultivars suggests that this function is possibly conserved across a wide range of genotypes. The *StPPO8* gene showed a differential expression pattern, with higher transcription levels in the local, purple-fleshed cultivars, particularly in Sapa. This observation would imply that the *StPPO8* gene may not be directly associated with the intensity of enzymatic browning, but rather is involved in other physiological processes, such as defense responses or the regulation of phenolic metabolism, functions previously described for other members of the PPO gene family [39,40,41,42]. 

Finally, gene expression in PPOs was evaluated in roots of the in vitro-grown potato plants. The rationale was that it was important to provide environment-controlled conditions for the analysis of gene expression among all genotypes. Although enzymatic browning mainly occurs in tuber tissues, PPOs are involved in phenolic metabolism and oxidative responses in different plant tissues [40,41]. Therefore, root expression patterns may happen during the early stages of potato tuber development [34]. We also tried to induce the production of micro-tubers under in vitro conditions but it was unsuccessful. 

Together, these findings provide a biochemical and molecular framework supported by experimental evidence observed in purple-fleshed potato cultivars. This study also identified candidate genes such as *StPPO2* and *StPPO8*, which may have potential use in future basic research but can be also applied in plant breeding programs that use genome editing [43].

## 4. Materials and Methods

### 4.1. Plant Material

Tubers were collected from three purple potato cultivars (*Solanum tuberosum* subsp. *andigena*), namely Siricha, Angashungo and Sapa, as well as from the white-fleshed cultivar Blanca. All of the plant materials originated from the districts of La Jalca and Luya, in the Amazonas Region, Peru. Authorization was granted by Directorial Resolution No. 013-2025-INIA-DGIA. The cultivar Desirée (CIP800048) was provided by the International Potato Center (CIP, Lima, Peru). Some of these tubers were stored at 4 °C for bioactive compound and colorimetric analyses, while the remaining tubers were cultivated under greenhouse with natural light conditions at the Toribio Rodríguez de Mendoza National University to obtain full-grown plants.

### 4.2. Preparation of Methanolic Extracts for Bioactive Compound Testing

Analyses were performed using raw potato tuber samples that were freeze-dried and subsequently ground to obtain a homogeneous powder. From this, 1.0 g of each sample was placed in clean test tubes. Bioactive compounds were extracted by adding 10 mL of 80% (*v*/*v*) methanol, followed by sonication in an ultrasonic bath for 10 min, ensuring that the solvent level remained within the effective ultrasonic range. An additional 10 mL of the same solvent was then added and sonication was repeated for a further 10 min. Subsequently, the samples were heated at 80 °C for 5 min to facilitate the extraction of soluble phenolic compounds. The extract was filtered through a 0.45 µm membrane to remove solid residues [44] and stored in amber bottles at 4 °C until further analysis.

### 4.3. Antioxidant Activity via DPPH Free Radical

Antioxidant capacity was determined using the 2,2-diphenyl-1-picrylhydrazyl (DPPH) (Sigma-Aldrich, St. Louis, MO, USA) free radical assay, following the methodology described by Brand-Williams et al. [45] with minor modifications for methanolic potato extracts. A methanolic DPPH solution of (20 mg/L) was prepared, with an initial absorbance of approximately 0.45 ± 0.02 at 516 nm. For the assay, 25 µL of the methanolic extract was mixed with 200 µL of the DPPH solution. The reaction mixtures were incubated in the dark for 10 min at room temperature (22 °C), and then the final absorbance (A_t_) was measured at 516 nm using a Varioskan LUX multimode microplate reader (Thermo Fisher Scientific, Waltham, MA, USA). All analyses were performed in triplicate, and the results were expressed as the percentage of DPPH radical inhibition, calculated using the following equation:

% *DPPH inhibition* = ((*A*_0_ − *A_t_*)/*A*_0_) × 100

where A_0_ is the initial absorbance of the DPPH solution and A_t_ is the absorbance after reaction with the extract. Antioxidant capacity was expressed as Trolox equivalents (µM TE/L), using a calibration curve constructed with standard Trolox concentrations ranging from 0 to 500 µM.

### 4.4. Determination of Total Phenol Content (Folin–Ciocalteu)

The total phenolic compound content was determined using the Folin–Ciocalteu method, following the methodology described by Çelik and Gökmen [46], adapted for reading in 96-well microplates. Briefly, 20 µL of the methanolic extract and 100 µL of Folin–Ciocalteu reagent (1:10 *v*/*v*) were added to each well, followed by the addition of 80 µL of 20% (*w*/*v*) Na_2_CO_3_, resulting in a final volume of 200 µL per well. The microplates were incubated at 50 °C for 5 min in the dark to allow the development of the characteristic blue color of the phenolic complex. Subsequently, the absorbance was measured at 765 nm in a Varioskan LUX multimode microplate reader (Thermo Fisher Scientific, Waltham, MA, USA). All analyses were performed in triplicate, and the results were expressed as milligrams of gallic acid equivalents per 100 g of sample (mg GAE/100 g), calculated from a gallic acid calibration curve (0–16 mg/L).

### 4.5. Colorimetric Analysis of Enzymatic Browning

For the evaluation of enzymatic browning, commercial tubers were used and cut into 1 cm thick slices under controlled conditions. Colorimetric measurements were recorded at 0, 2, 6 and 24 h after cutting, obtaining the L*, a*, and b* values corresponding to lightness (L*), red–green component (a*), and yellow–blue component (b*). These time points were selected to capture the early, intermediate, and advanced stages of enzymatic browning following tuber cutting. Measurements were performed using a CR-400 colorimeter (Konica Minolta, Osaka, Japan), previously calibrated with a standard white reference plate. Based on the data obtained, the EBI was calculated using the equations proposed by Coklar et al. [47] to quantify the intensity of tissue darkening over time.

(1) $$X=\frac{\left(a*+1.75\right)}{\left(5.642L*+a*-3.012b*\right)}$$

(2) $$EBI=\frac{\left(X-0.31\right)}{0.172}\;100$$

### 4.6. In Vitro Establishment of Potato Cultivars

Plants grown under greenhouse conditions were used as a source of explants for in vitro establishment. Apical buds were previously disinfected following the methodology described by Hajare et al. [48] to eliminate potential external contaminants. Meristems were isolated from the apical buds under a stereomicroscope, preserving only the active meristematic region. The isolated meristems were cultured in Murashige and Skoog (MS) medium supplemented with 30 g L^−1^ sucrose. The pH of the medium was adjusted to 5.7 prior to the addition of agar (6 g L^−1^) and subsequent sterilization. Cultures were incubated at 22 °C under a photoperiod of 16 h light and 8 h dark, with a light intensity of 60 µmol m^−2^ s^−1^, relative humidity of 25%, and 10% ventilation in a growth chamber [49,50]. The in vitro seedlings obtained were used as experimental material for gene expression analyses.

### 4.7. Obtaining Potato Minitubers

Two-month-old in vitro plants of all cultivars (Desiree, Sapa, Siricha, and Angashungo) were used as starting material for the mini-tuber production under greenhouse with natural light conditions. The acclimatization process followed the technical protocol for pre-basic potato seed production [51], with modifications adapted to the conditions of this study. Seedlings were gradually released from the culture medium through progressive dilution with distilled water, with daily solution changes and exposure to low light, until all gel residues were completely removed (4–5 days).

Subsequently, the roots were briefly immersed in a Root-Hor^®^ rooting hormone solution (1 mL/L) and planted in germination trays containing sterile substrate composed of coconut fiber and peat (pH ~5.5). The seedlings were allowed to establish for 15–20 days under low light conditions and controlled humidity. Once vigorous growth was observed, the plants were transferred to the greenhouse and transplanted into a substrate previously fertilized with NPK (20-20-20). The plants were set at a depth of 5 cm and spaced at 15 × 15 cm. Crop management included localized irrigation every four days, hilling at 30 and 60 days after planting, and regular applications of plant protection products (Atak^®^ and Evisec^®^, 1 g/L) at intervals of 15–21 days.

### 4.8. Biochemical Determination of the Enzymatic Browning Index in Mini-Tubers

The evaluation of enzymatic browning was carried out following the protocol described by Chi et al. [33], with minor modifications. For this analysis, mini-tubers derived from in vitro plants and grown in a greenhouse were used. The samples were cut into 500 mg sections of fresh tissue and immediately frozen in liquid nitrogen and ground to a fine powder, which was homogenized with 2 mL of cold extraction buffer consisting of 100 mM sodium phosphate (pH 6.0), 2% Triton X-100, and 2% PVPP. The extracts were allowed to oxidize for one hour at room temperature and then centrifuged at 11,000 rpm for 10 min in 1.5 mL microcentrifuge tubes. The absorbance of the supernatant was measured at 475 nm (A_475_) using a Varioskan LUX (Thermo Fisher Scientific, Waltham, MA, USA) 96-well multimode microplate reader, with 200 µL per sample and three technical replicates.

Total protein concentration was determined using the Pierce BCA Protein Assay kit (Thermo Fisher Scientific, Waltham, MA, USA) using the same equipment. The enzymatic browning index (EBI) was expressed as the ratio A_475_/mg total protein.

### 4.9. Extraction and Quantification of Total RNA

Root tissues from in vitro cultivars of Siricha, Angashungo, Sapa, and Desireé were ground in liquid nitrogen using a pre-cooled mortar. Total RNA was extracted from the pulverized material using PureZOL™ RNA Isolation Reagent (Bio-Rad, Hercules, CA, USA), following the manufacturer’s instructions. The resulting RNA pellet was resuspended in 30 µL of RNase-free water and stored at –20 °C until further use. RNA concentration and purity were determined by measuring 1 µL of each sample in a SmartDrop NS1000 (Thermo Fisher Scientific, Waltham, MA, USA). Concentration was expressed in ng/µL, and RNA quality was assessed based on the A_260_/A_280_ absorbance ratio.

### 4.10. cDNA Synthesis

The first-strand cDNA was synthesized from total RNA extracted from in vitro plant roots using the GoScript™ Reverse Transcription System kit (Promega, Madison, WI, USA). Up to 5 µg of RNA per reaction was used along with Oligo(dT) oligonucleotides. The RNA–primer mixtures were incubated at 70 °C for 5 min and then cooled on ice. The reverse transcription reaction mix was subsequently added, bringing the final volume to 20 µL. Reactions were incubated at 25 °C for 5 min, 42 °C for 1 h, and 70 °C for 15 min to inactivate the enzyme. The resulting cDNA was stored at −20 °C and used as a template for gene expression analysis by PCR.

### 4.11. Semi-Quantitative Analysis of Gene Expression Using PCR

Gene expression analysis was performed using cDNA derived from in vitro plant roots. Amplifications were carried out by polymerase chain reaction (PCR) using DreamTaq™ DNA Polymerase (Thermo Fisher Scientific, Waltham, MA, USA), following the manufacturer’s instructions. The elongation factor 1 alpha (ef1α) gene was used as a reference for cDNA normalization for each sample for 20 cycles at an annealing temperature of 56 °C. Subsequently, the genes encoding polyphenol oxidase (PPO) were amplified under the same conditions, with the program adjusted to 28 cycles and an annealing temperature of 58 °C (Table 1).

PCR reactions were carried out with an initial denaturation at 95 °C for 2 min, followed by cycles of 95 °C for 30 s, 58 °C for 45 s, and 72 °C for 30 s, concluding with a final extension at 72 °C for 5 min and storage at 4 °C. The amplified products were separated by electrophoresis on a 2.5% agarose gel, stained with Diamond™ Nucleic Acid Dye (Promega, Madison, WI, USA), and visualized under ultraviolet light. A 1 kb Plus DNA Ladder (Thermo Fisher Scientific, Waltham, MA, USA) was used as a standard molecular weight marker to verify the expected size of the amplicons. 

### 4.12. Quantitative Analysis of Gene Expression Using Digital PCR

Reactions were carried out in the QIAcuity system (QIAGEN) using EvaGreen intercalating dye, following the manufacturer’s recommended protocols for EvaGreen-dPCR assays. Each reaction mixture had a final volume of 40 µL, consisting of 13.3 µL of 3x EvaGreen PCR Master Mix (QIAGEN GmbH, Hilden, Germany), 1.6 µL of each primer (forward and reverse) at a working concentration of 10 µM, 13.5 µL of RNase-free water, and 10 µL of cDNA. The reactions were loaded into 24-well plates of the QIAcuity system, each containing approximately 26,000 active partitions enabling high-resolution absolute quantification. Prior to analyzing the genes of interest, quantification was standardized using the constitutive EF-1α gene to determine the optimal cDNA dilution, ensuring an appropriate number of positive and negative partitions within the recommended dynamic range for dPCR.

The thermal program began with an initial activation at 95 °C for 2 min, followed by 40 cycles consisting of denaturation at 95 °C for 15 s, annealing at 58 °C for 15 s, and extension at 72 °C for 15 s, concluding with a final hold at 40 °C for 5 min.

An analysis of positive and negative partitions, as well as the determination of absolute gene concentration values expressed as copies/µL, was performed using QIAcuity Software Suite 3.2 (QIAGEN, Hilden, Germany). For comparison between samples, PPO gene expression levels were normalized to the ef1α reference gene and expressed as relative percentages.

## 5. Conclusions

Native purple-fleshed potato cultivars were shown to have a distinctive metabolic profile, which was characterized by a higher phenolic compound content and greater antioxidant capacity compared to white-fleshed cultivars. These biochemical characteristics were consistently associated with increased susceptibility to enzymatic browning, as evidenced by the data collected in complementary colorimetric and chemical analyses. This supported a close relationship between phenolic substrate availability and the intensity of oxidative processes.

At the molecular level, an analysis of the *StPPO* gene family identified *StPPO2* and *StPPO8* as the predominantly expressed isoforms in roots, with *StPPO8* standing out due to its preferential expression in local purple-fleshed cultivars compared with the reference cultivar Desirée.

All of these findings obtained through biochemical and molecular evidence provided a comprehensive understanding of the relationship between *StPPO* gene expression and enzymatic browning in purple-fleshed potatoes. Future research could explore knockouts of specific PPO genes as a strategy to reduce browning while preserving the nutritional and bioactive compound profile of these cultivars.

## Figures and Tables

**Table 1:** Primer sequences used for amplification of the reference gene and polyphenol oxidase (PPO) genes, described by [14].

Gene Name	Gene ID	Primer Sequences	Annealing Temp. (°C)
		(Forward/Reverse, 5′-3′)	
*StPPO1*	PGSC0003DMG4000295751	TTGACACACCTCAGCTCCAGA	
		GTAAGCAGCACCGAAGAATTG	58
*StPPO2*	PGSC0003DMG400018916	ATATCGCGACTGTTGATTTCC	
		GTCGCACCTTCAATGGAGATA	58
*StPPO3*	PGSC0003DMG400018914	ATGGCGTAACTTCAAACCAAA	
		CCATCTTCGTGAGTGGGAATA	58
*StPPO4*	PGSC0003DMG400018917	TCTGGTGCCAAAGAAAGGTAA	
		ACAAACAATCCGCAGATTCAA	58
*StPPO5*	PGSC0003DMG400018919	ACTATGCGGGAAAAGAAGGGA	
		CTGGCGCGTAATCATAACCC	58
*StPPO6*	PGSC0003DMG400029576	GGCTTTTCTTCCCGTTCCAT	
		GGAGGTAAACGCATGCCTTT	58
*StPPO7*	PGSC0003DMG400018924	CCTCATACTCCGGTCCACAT	
		CGGCTGAGTAGAAATTGCCC	58
*StPPO8*	PGSC0003DMG400018913	ATTCGCGGTATGGGTACGAT	
		TGGGATCTCTTGCAGCTGAA	58
*StPPO9*	PGSC0003DMG400022430	GGACCCGACGTTACCAAATG	
		TGATGGAAGCTGGAAGTCGA	58
*StPPO10*	PGSC0003DMG400018925	AAAGTTTTCACGTCTCATGC	
		AAACACTATAGAGCCCTCCT	58
*ef 1α*	-	ATTGGAAACGGATATGCTCCA	
		TCCTTACCTGAACGCCTGTCA	56

## Data Availability

The data presented in this study are available upon request from the corresponding author. The data are not publicly available as they support ongoing analyses and future publications.

## Abbreviations

The following abbreviations are used in this manuscript:

PPOs	Polyphenol oxidases
DPPH	2,2-diphenyl-1-picrylhydrazyl
EBI	Enzymatic browning index
